# Perceptions and experiences of perinatal mental disorders in rural, predominantly ethnic minority communities in northern Vietnam

**DOI:** 10.1186/s13033-016-0043-0

**Published:** 2016-02-24

**Authors:** Daniel Abrams, Liem T. Nguyen, Jill Murphy, Younji (Angie) Lee, Nhu K. Tran, David Wiljer

**Affiliations:** University of Toronto, Toronto, Canada; Institute of Population, Health and Development, Hanoi, Vietnam; Simon Fraser University, Burnaby, Canada; Dartmouth College, Hanover, USA; Centre for Addiction and Mental Health, Toronto, Canada

**Keywords:** Vietnam, Mental health, Perinatal, Primary care, Community health

## Abstract

**Background:**

Preliminary research has suggested that perinatal mental disorders (PMDs), including post-partum depression, are prevalent in Vietnam. However the extent to which these disorders are recognized at the community level remains largely undocumented in the literature. PMDs have also never been investigated within Vietnam’s significant ethnic minority populations, who are known to bear a greater burden of maternal and infant health challenges than the ethnic majority.

**Objective:**

To investigate knowledge and perceptions of PMDs and their treatments at the community level in a rural, predominantly ethnic minority region of northern Vietnam.

**Methods:**

Qualitative semi-structured interviews were conducted on the topic of common PMDs. Participant groups were primary health workers (PHWs) working at local community health centers, and pregnant or postpartum women enrolled in a program for maternal and infant health that was not mental health related. Interviews included vignette scenarios that asked respondents to interpret cases of women experiencing PMDs, as well as open-ended questions about mental disorders and their treatments.

**Results:**

Twelve PHWs and 14 perinatal women completed the study. Major themes that emerged from the interviews included (1) Family relationships impact psychological well-being, (2) Nutrition contributes to perinatal mental health, (3) Both traditional and western medicine play roles in perinatal health, (4) There was a lack of personal experience with women experiencing PMDs, (5) Descriptions of mental health symptoms focused on behaviours, and (6) Community care is the primary mental health support.

**Conclusions:**

PHWs reported having almost never treated a woman with a PMD. However, anecdotal evidence from the women interviewed suggests that there are incidents of mental disorders during the perinatal period that go largely unaddressed. Willingness to present to primary care appears to be high, and presents an opportunity to address this need by training PHWs in effective screening, treatment, and referral. Such training should account for culturally specific presentations of mental disorders as well as the importance of the patient’s social context. To the best of the author’s knowledge, this research presents the first evidence of a PMD burden within Vietnam’s ethnic minority communities.

## Background

Common mental disorders, which are defined as non-psychotic mental health conditions and include depression and anxiety [1], have been associated with significant functional disability across cultures [2]. Perinatal mental disorders (PMDs) refer to common mental disorders that occur during pregnancy or in the first year postpartum. The incidence of perinatal depression in western countries is estimated at 13 % [3], while estimates of perinatal generalized anxiety have ranged from 2.6 to 29 % [4].

Evidence of PMD prevalence and impacts in lower-middle-income countries (LMICs) is emerging but incomplete, with many countries represented by only one peer-reviewed paper in the English-language literature [5]. One systematic review and meta-analysis found that 16 % of pregnant women and 20 % of women in the postpartum period screen positively for PMD symptoms [5]. Risk factors for PMD that occurred across many settings included socioeconomic disadvantage, quality of the relationship with an intimate partner and with in-laws, and preference for male children [5]. Another review of postpartum depression (PPD) specifically in Asian countries found prevalence ranging from as low as 3.5 % in Malaysia to as high as 63.3 % in Pakistan [6].

Preliminary research into PMDs in Vietnam has made use of a variety of screening tools and revealed prevalence rates ranging from 16.9 [7] to 39.9 % [8]. It appears that PMDs are more prevalent in rural populations than in major urban centres [9, 10]. As in other settings, PMDs appear to have multiple deleterious impacts on both maternal and infant health in Vietnam. Vietnamese women who screen positively for PMD symptoms may be less adherent to perinatal healthcare recommendations [7]. In one population of infants in rural settings, maternal PMD during pregnancy was found to have a negative impact on motor development at 6 months [8]. Symptoms of antenatal depression during the third trimester have been associated with low birth weight and preterm birth [11], as well as child stunting [12].

While there is an emerging picture in the English language literature that suggests that PMDs in Vietnam are prevalent and have adverse affects on maternal and infant health, there are few policies for the provision of mental health care outside of hospital-based care of patients with severe mental illness [13]. The majority of patients seen at the country’s mental health hospitals are treated for psychotic disorders and epilepsy [14]. PMDs and other common mental disorders were not addressed by Vietnam’s official mental health policy until 2010, when depression was added [13, 15]. This update also targeted the integration of mental health services into primary care [15]. Vietnamese patients without private health insurance can only access specialist care via referral from their assigned primary care centre, known in Vietnam as a commune health centre (CHC). Primary health care workers at CHCs are thus the essential gatekeepers to all mental health services for these patients. During literature review no data was retrieved indicating the present success of primary care integration into mental health, however ongoing research suggests that CHCs have minimal capacity to screen for PMDs or to provide pharmacotherapeutic or psychosocial treatments for PMDs.

The scarcity of data on the detection and treatment of PMDs in Vietnam is further complicated by the fact that there may be important cultural differences in the conception of mental health itself between those writing in the English-language literature and Vietnamese patients and practitioners in the community. Vietnamese conceptions of mental health are influenced by Confucianism and Buddhism, and include a more holistic view of the mind–body relationship [16]. In this view psychological disturbance is linked to physical disturbance, and there is no typology of independent mental disorders as in western psychiatry [16]. While literature suggests that adapted instruments for the detection of PMDs yield positive findings [17, 18], there is still no consensus as to whether the disease states they are designed to detect represent meaningful constructs for the majority of Vietnamese patients and practitioners. Ongoing, unpublished work in this area suggests that ‘mental illness’ is colloquially understood as pertaining only to sever psychosis by many in Vietnam. Others have argued that attempts to encompass wide cultural differences in the experience and perception of mental illness within a single international taxonomy may create a misguided impression of cross-cultural validity for western-derived diagnostic systems [19].

Absent from the current English-language literature in maternal mental health are Vietnam’s numerous ethnic minority groups, who together comprise 14.2 % of the country’s population [20]. On average, ethnic minority Vietnamese are subject to more adverse maternal and child health outcomes than the majority [21] including significantly increased rates of infant mortality and under-five mortality [20]. Women from ethnic minority groups are known to make less use of maternal health care services, which may be due in part to relative socioeconomic disadvantage as well as relative isolation from health services [22]. At present it remains undocumented whether mental health is a contributor to these disparities.

The present study attempts to understand how PMDs are currently perceived and addressed at the community level. Using semi-structured interviews, this study engaged women and primary health care providers in rural, primarily ethnic minority communities in conversations about their experiences and perceptions of PMDs.

## Methods

### Setting

This study was carried out in four communities located within the Dinh Hoa district of Thai Nguyen province. Dinh Hoa is a rural, mountainous district with a large ethnic minority population. It is located in Vietnam’s northern Midland and Mountain region: the only region in which ethnic minority groups outnumber the Kinh ethnic majority according to a 2009 census [21].

Fieldwork for this study was implemented as a part of the project ‘Improving maternal and perinatal care for ethnic minorities in Thai Nguyen, Vietnam through an integrated eHealth and user-provider interaction model’, or mMOM, which is co-led by the Institute of population, health and development (PHAD) and Thai Nguyen provincial health department (TNHD). The mMOM project is funded by the Canadian international development research centre (IDRC), implemented in Dinh Hoa district, and received ethical approval from the internal review board of PHAD.

### Participants and recruitment

Primary health workers (PHWs) were recruited from the CHCs of each of the four communities included in the study. Inclusion criteria comprised PHWs who would regularly provide care for pregnant and postpartum women. This included physicians, physician’s assistants, midwives, nurses, and pharmacists.

Women who were either pregnant or in their first year post-partum (herein referred to as ‘Mothers’) were recruited from the communities served by the same CHCs used for PHW recruitment. The women were contacted first by local PHWs and pre-screened for their availability and willingness to participate in the study.

All participants were literate and provided written consent for participation in the study. Participants were also given the opportunity to opt out of having the interview audio recorded.

### Interview content

The semi-structured qualitative interviews began with demographic information including ethnicity, employment, educational background, and family composition.

Participants were then asked to respond to two vignette scenarios designed to illustrate cases of women who may be suffering from a PMD. The participants were not told that the interview was mental health related so as not to bias their responses. The use of vignettes to prompt unbiased responses to mental disorders was inspired by a previous investigation into explanatory models of depression in another region of Vietnam [23]. Scenario 1 referred to a woman with possible post-partum depression (Table 1), and scenario 2 referred to a woman with possible antenatal generalized anxiety (Table 2). The scenarios were designed to encompass diagnostic criteria from the diagnostic and statistical manual of mental disorders IV (DSM-IV) [24] with specific descriptive statements borrowed from the Phan Vietnamese psychiatric scale (VPS) [25]. The vignettes were written in English, translated into Vietnamese, and then back-translated to English by a second translator to check for conceptual equivalence [26].

**Table 1:** Postpartum depression English text

Scenario 1	
"A 24 year-old woman, a new mother of a 2-month old infant, complains of headaches and trouble with sleep that has lasted for the past 4 weeks. She finds that she often wakes during the night, and cannot sleep well even when the baby sleeps. She does not have a fever. Her speech and movement are slower than normal. She says that she has lost her appetite, and does not enjoy eating as much as she used to. She feels ashamed because she has difficulty performing her usual activities and caring for her child, and her family has expressed concern about her behaviour. She admits that she does not breastfeed her child often. She feels worthless and sometimes thinks that she does not want to live anymore."

**Table 2:** Antenatal generalized anxiety disorder English text

Scenario 2	
"A 23 year-old woman, 7 months pregnant, is brought to the clinic by her concerned sister. The woman has been experiencing dizziness and feelings of weakness in her limbs. She has become fearful for the health of her unborn baby, and is unwilling to leave the vicinity of her home. She has experienced several episodes where she feels unable to breathe and begins to feel either extremely hot or cold. Occasionally she experiences heart palpitations. She visited the hospital during one of these episodes but the doctors were unable to identify a cause."	

Both mothers and PHWs were asked to interpret the scenarios using questions inspired by Kleinman’s illness explanatory model framework [27]. This included asking for participant insights into whether they felt the woman in the scenario was experiencing a medical problem, what kind of problem it might be, what may have caused the problem, and what supports may be available.

Following the vignette scenario interpretation, participants were informed that the purpose of the interview was to discuss perinatal mental health. Participants were then asked more explicitly about their experiences and perceptions of PMDs. For example, PHWs were asked about their clinical experience with PMDs, and mothers were asked for their perceptions of maternal mental health issues in the community. All questions related to mental health were phrased in the abstract, and mothers were not asked about their own personal mental health histories.

### Data collection and analysis

All interviews were conducted in Vietnamese, and the interview team consisted of at least one bilingual Vietnamese/English speaker and one English-only speaker. The bilingual researcher was not a certified translator, but met the criteria of ‘sociolinguistic language competence’ defined as the ability to communicate between languages using complex sentence structures, a high level of vocabulary, and the ability to describe concepts or words when they do not know the actual word or phrase [26, 28]. The bilingual researcher conducted the interview in Vietnamese, and provided translation for the English-speaking researcher. Both interviewers acted as ‘active producers of research’ by generating follow-up questions for participants [29], and both kept their own set of field notes during the interview. Interviews were also audio recorded when consent could be obtained to do so.

Interviewers later met to compare their individual field notes and integrate them into a single final set of notes for each interview. Participant comments were coded by key terms or phrases and then organized into themes and sub-themes by two members of the research team (DA and DW) using a grounded theory approach [30]. Mothers and PHWs responses were analyzed in separate groups but through use of the same coding scheme so that common themes could be identified. The analyses were compared for consistency and amended until a consensus was reached on final themes and sub-themes.

## Results

### Participants

Twelve PHWs participated in the study. Eight of the 12 workers were female and nine were of the Tay ethnic minority (Table 3). A variety of medical professions were represented, with the most common being doctor’s assistants. Their median age was 44 years, and their age range was 24–60. The mean number of years of work experience at the CHC was 13 years.

**Table 3:** PHWs demographics

Characteristic	No. of participants
*Sex*	
Male	4
Female	8
*Education*	
Medical university	3
Vocational college	5
Intermediate	2
High school	1
*Ethnicity*	
Tay	9
Kinh (majority)	3
Job role	
Doctor	3
Doctor’s assistant	5
Midwife	2
Pharmacist	1

Fourteen mothers completed the interview. Twelve of the 14 participants were currently pregnant, with fewer mothers in their first year postpartum were recruited (Table 4).

**Table 4:** Mothers’ demographics

Characteristics	No. of participants
*Perinatal status*	
Pregnant	12
Postpartum	2
*Ethnicity*	
Tay	6
Sau Chi	1
Dao	1
San Tri	1
Kinh (majority)	5
*Education*	
College	3
High school	3
Secondary school	8
Average household composition	
Adults	3
Children	1

Nine of the mothers were from ethnic minority groups, with the most represented group being the Tay. Mothers reported their mean yearly household income to be 45.1 million VND ($2100 USD). The median age of the mothers was 25 and their age range was 21–36.

### Scenario 1 was often perceived to be describing a mental health problem

The majority of PHWs and half of mothers used language related to mental health to label the problem that the woman in scenario 1 was experiencing. A few used the label ‘depression’ explicitly, while others used descriptors such as ‘a mental health problem’ or ‘a psychological problem’.

### Scenario 2 was often perceived to be a circulatory or cardiac problem

A majority of PHWs identified the central problem in scenario 2 as being related to circulatory or cardiac dysfunction, and did not use language related to mental health. Specific problems identified included ‘anemia’, ‘hypertension’, ‘hypotension’, and ‘a lack of blood flow to the brain’. Three PHWs labeled the problem as ‘neurasthenia’, a label that has emerged in other studies of mental health in Vietnam and China [31]. A significant minority of mothers and one health worker felt that the symptoms described in this scenario do not represent a health problem during pregnancy.

### Themes identified in the interviews

The six major themes identified in analysis of the interview notes were as follows:

1. Family relationships impact psychological well-being.
2. Nutrition contributes to perinatal mental health.
3. Both traditional and western medicine play roles in perinatal health.
4. Lack of personal knowledge of women experiencing PMDs.
5. Descriptions of mental health symptoms focused on behaviours.
6. Community care is the primary mental health support.

Mother’s and PHW’s responses are presented together within each theme, with comments on the agreements or disagreements in responses that emerged between groups.

#### 1. Family relationships impact psychological well-being

Most mothers and all PHWs offered that stressful family relationships could contribute to the development of symptoms like those listed in the two scenarios. Some PHWs and mothers specified that since most women move in with their husband’s families after marriage, the relationships between women and their in-laws could be particularly stressful.

All PHWs and nearly all mothers also cited family members as important sources of support for women experiencing mental health challenges. The family was cited as the single most important source of support, more important than formal medical care, by a majority of respondents in both groups.

Husbands were mentioned more than any other specific family member both as potential sources of psychological stress as well as support. One example of relationship stress cited by respondents in both groups is that in the communities surveyed many husbands take jobs outside the community, which keep them away from home for long periods of time.

#### 2. Nutrition contributes to perinatal mental health

A majority of PHWs and mothers mentioned that improper nutrition could be a contributor to poor mental health during the perinatal period, and that nutritional counseling should be part of a treatment plan. While the interviews did not retrieve rich information on specific nutritional advice, some examples offered included ‘she should eat more’, as well as the inclusion of herbal ingredients used in traditional medicine.

#### 3. Both traditional and western medicine play roles in perinatal health

Some PHWs suggested that traditional herbal medicines are safer than pharmaceuticals for use during the perinatal period. One stated that he would try traditional medicines first, since they have fewer side effects, and consider Western medications only if these failed.

Few respondents mentioned the possibility of pharmaceutical medication to treat the women in scenarios 1 or 2. Some PHWs and mothers stated that it is unsafe for women who are pregnant or breastfeeding to use western pharmaceutical medications at all. One worker related an anecdote of a local pregnant woman with a pre-existing psychiatric condition who was taken off medication during pregnancy and experienced a serious deterioration of her mental health as a result.

Some PHWs, but no mothers, also mentioned traditional folk beliefs that ‘ghosts’ or ‘spirits’ are the cause of mental health problems. Of those who mentioned these beliefs, some PHWs felt that ‘people thought like this 20 years ago, but no one believes this today’. Others felt that some in community still actively hold these beliefs, and that individuals who live far away from the CHC might seek help from a traditional shaman before seeking medical help.

#### 4. Lack of personal knowledge of women experiencing PMDs

Few PHWs reported having ever seen a woman experiencing a mental health disorder during the perinatal period. PHWs’ experience with mental health patients of any kind was reported to be mostly limited to the long-term management of patients with psychosis or epilepsy, and very few reported diagnosing a new mental health patient within the past year.

While over half of mothers also reported having no first hand knowledge of women experiencing PMDs, a few did report such experience. Some mothers related cases of other women they knew in the community and three mothers told interviewers that they themselves could relate to the women described in the scenarios. One woman expressed her frustration that she had previously visited her local CHC, but did not feel like bringing up her problems because it was ‘too crowded’ and the health care providers ‘did not ask about her feelings’. She says she was told that she is ‘weak’ and ‘tired’. This woman said that she would like to see a psychologist but doesn’t know how to get a referral.

Another woman described how her anxiety and insomnia has returned since being told to go off her medication during pregnancy, and that she has been told she ‘lacks blood flow to the brain’ and should take iron pills. A third woman reported that she had helped herself through postpartum depression in the past after learning about depression through TV programs and magazines.

#### 5. Descriptions of mental health symptoms focused on behaviours

When asked what types of symptoms they expected patients with PMDs to experience, all PHWs and most mothers mentioned abnormal behaviour and speech. Specific examples included ‘wandering around outside’, ‘attacking someone’, and ‘speaking without meaning’. Changes in sleep and appetite were also mentioned.

Mothers, however, were more likely than PHWs to refer to thoughts or emotions when speaking about mental health. The most commonly mentioned symptoms in this category related to worry, anxiety, or stress.

#### 6. Community care is the primary mental health support

Almost all PHWs and the majority of mothers felt that if a woman were experiencing mental health problems during the perinatal period she would seek help from the CHC either on her own or by being taken there by family members. Most PHWs stated that they would provide advice to a woman experiencing mental health problems, for example counseling her on rest or nutrition. Only one PHW reported having ever received training that mentioned perinatal mental health, and none made reference to guidelines for counseling women during the perinatal period. One physician stated that he would tell the woman in scenario 1 that she was ‘doing the wrong things’.

Several PHWs mentioned that mental health cases are often referred to a higher level hospital where more specialized care is available.

## Discussion

### PMD incidence and detection

Both the Mothers’ and PHWs’ responses to scenario 1 suggest that they might be likely to recognize a woman with postpartum depression as having a problem related to psychology. However, the reflection from the majority of PHWs that they had never diagnosed or treated a woman with a PMD, despite the literature suggesting PMDs are prevalent in rural Vietnamese communities [5, 7, 9, 10], is less encouraging. The fact that mothers were more likely than PHWs to be able to relate a case of a woman experiencing mental health challenges during the perinatal period further suggests that there is a burden of PMDs in these communities going largely unrecognized by primary care providers.

Of particular interest were the anecdotes provided by three women who volunteered that they have suffered from depressive symptoms either currently or during a past pregnancy. Although they were asked no direct questions about their own health history, the story in scenario 1 prompted these women to share their own similar experiences with the interviewers. One woman related her experience of seeking help at the CHC, but finding that PHWs there did not attend to her emotional state. Interestingly, the workers interviewed at that same CHC reported having never met a woman with a PMD, however we cannot confirm whether the PHWs interviewed in this study were the same ones who had seen this woman clinically. In her case, what she needed was for the PHWs to proactively ask about her mood and emotional state. This may be a valuable insight for the future improvement of PMD screening in similar communities.

The responses to scenario 2 suggest that a patient presenting with generalized anxiety as described above is likely to be misdiagnosed with a disorder of the circulatory or nervous system. Since depression is now included in Vietnam’s national mental health plan while anxiety is not, the discrepancy in responses observed here may be a reflection of the fact that PHWs have received some training in depression detection in reinforcement of that policy. It may also be the case that the vignette used here was not a realistic example of a patient presentation in a Vietnamese context. While our vignette was based on culturally specific descriptions of symptoms listed in the VPS [25], manifestations of anxiety in Vietnamese patients are not as well corroborated in the literature as are manifestations of depression. More research will be needed to delineate the extent to which perinatal anxiety disorders are present in Vietnam and how they might commonly be described.

When discussing the expected symptoms of a patient experiencing a PMD, behavioural symptoms were discussed more often than internal thoughts, feelings, and emotions. This fits the findings of others that Vietnamese patients with depression primarily report their experience in the context of somatic symptoms such as insomnia and headaches, and reinforces the need for the development of culturally acceptable screening measures. It is also notable that the behaviours cited by participants were often more suggestive of psychotic disorders than common mental disorders like depression and anxiety. This indicates the difficulty of approaching mental health in Vietnam based on the taxonomy of mental disorders as defined by the DSM-V. Due to traditional cultural conceptions of mental health as a singular entity inextricably tied to physical health, as well as government mental health policy which only explicitly addressed schizophrenia and epilepsy prior to 2010, conceiving of PMDS in diagnostic categories wholly separate from psychosis may not be intuitive to many primary care providers, patients, and family members.

### The impact of personal social context on PMDs

Family relationships emerged strongly as both potential risk factors for PMDs and as important sources of support. The relationship between the women and their husbands was mentioned most often, which is in accordance with previous research that has found that the quality of the relationship with an intimate partner contributes both to likelihood of experiencing a PMD [5] and the rate of recovery from a PMD [32]. The present study also highlighted social and economic factors that may be risk factors for PMDs in these communities—namely the cultural norm that women will move in with their in-laws after marriage, and that husbands often leave home for extended periods of time in order to find work.

The emphasis placed on these factors by participants indicates that PMDs may not be considered as isolated biomedical conditions but rather as manifestations of disorder in a woman’s overall social, cultural, and economic situation. These findings highlight the importance of training PHWs to not only recognize symptoms of PMDs but also to investigate the stresses and supports available to women, in particular intimate partner relationships.

While most respondents identified the CHC as one resource for mental health support, the majority identified family support as taking priority over formal health care services. The family as the primary source of support for women potentially creates a problem for those women under psychological stress due in part to those same family relationships. As part of any assessment and support offered by the CHC it will be important to question assumptions as to whether or not a particular family can currently act as an effective support or whether they may in fact be contributing to mental distress.

### PMDs and ethnic minority communities

The majority of respondents, both mothers and PHWs, identified as belonging to an ethnic minority group with Tay being the most commonly identified ethnicity. Data from the government of Vietnam suggest that the Tay lag significantly behind the Kinh ethnic majority on maternal and child health indicators such as infant and under-five mortality [20]. However there are other ethnic groups, for example the H’mong, whose health outcomes are reported to be far worse [20]. This study provides the first direct evidence known to the authors that PMDs may represent an under-addressed health need for women in ethnic minority communities. However, it remains to be established whether the extent of this need differs from the ethnic majority and contributes to the observed disparities in maternal and child health.

### PMD treatment: present situation and future opportunities

In spite of the fact that PHWs may not currently receive the necessary training to detect PMDs [14], most respondents asserted the belief that patients would turn to the CHC as a primary resource for mental health support. The willingness of mothers to seek formal medical help was stronger in this sample than in a previous report conducted with a similar method at a district hospital in a semi-urban setting [23]. The present findings suggest that shortfalls in addressing PMDs at the community level may rest more with detection and diagnosis than with patient willingness to present. If so, this presents an opportunity to improve the wellbeing of a great number of Vietnamese women during the perinatal period by initiating training to bolster PHWs abilities to detect, treat, or make appropriate referrals for PMDs.

Our findings reinforce the need for training at the CHC level that is culturally relevant and takes account of patients’ broader socioeconomic context. As noted by others [31], the degree to which descriptions of mental health disorder rely on behavioural symptoms raises questions about the effectiveness of PMD treatments based on cognitive behavioural therapy, which demands the ability and willingness to discuss private experiences and emotions [33]. Family relationships and nutrition were two topics that proved to be particularly salient in the present study and should be addressed by future protocols for PMD detection and treatment.

Our interviews also touched upon the importance of traditional medicine to many Vietnamese patients as well as mainstream health practitioners. Vietnam is one of a few countries in which traditional medicine is integrated into the national health strategy as a regulated health profession with its own degree programs and certifications, and is available alongside biomedical care at many clinics and hospitals [34]. Some of the health providers interviewed in this study, none of whom were traditional medicine practitioners themselves, clearly regarded traditional medicine as an important resource for perinatal health and perhaps preferable to biomedical treatment for pregnant and breastfeeding women. It bears further investigation whether there are situations in which this belief may put women at risk by limiting their access to effective and safe medications during the perinatal period.

### Limitations

Recruiting postpartum women to participate in interviews proved more difficult than expected, and as such our sample consisted mostly of pregnant women. Part of the reason for this difficulty may be a tradition practiced in Vietnam, as well as China and Taiwan, that dictates that a woman should stay at home and rest for 30 days after giving birth, and may have limited contact with non-family members during this time [35].

The Mothers who participated in these interviews were also drawn from a sample population already engaged in a separate and ongoing intervention for the improvement and maternal and child health. While the current intervention does not address mental health, their engagement alone may have increased their perception of the CHC as a health support. Mother’s knowledge of CHC services and willingness to seek help may also have been influenced by above average educational attainment of women in this sample as compared to their communities, will all women interviewed having at least completed secondary school (Grade 9 equivalent). The authors were unable to obtain reference data specific to women of child bearing age in Dinh Hoa province, however a 2009 Vietnamese government analysis reported the secondary school enrolment rate for eligible children in all rural areas at only 80.7 % [36]. Current engagement with their CHCs and above average education may limit the generalizability of some findings.

The interviewing method of this study relied on in-person translation and field notes for the recording of participant responses. This precluded the research team from obtaining verbatim quotes, and subtleties of meaning or emphasis may have been lost in the translation process. The interview team addressed this limitation through extensive debriefing and comparison of notes between the Vietnamese-speaking and non-Vietnamese speaking researchers who were present at each interview.

## Conclusions

Primary healthcare workers in four rural, predominantly ethnic minority communities report having almost never treated a woman with a PMD. However, anecdotal evidence from women in these communities suggests that there are incidents of mental disorder during the perinatal period that go largely unaddressed. Willingness to present to primary care appears to be high, and presents an opportunity to address this need by training PHWs in effective screening, treatment, and referral. Such training should account for culturally specific presentations of mental disorders as well as the importance of the patient’s social context. These interviews provide the first direct evidence of a burden of PMDs in Vietnam’s ethnic minority communities; more research is needed to establish whether PMDs contribute to the maternal and child health disparities faced by these groups.

## Abbreviations

CHC: commune health centre
DSM-IV: diagnostic and statistical manual of mental disorders IV
IDRC: International development research centre
LMIC: lower-middle income country
PHAD: Institute of population health and development
PHW: primary health worker
PMD: perinatal mental disorder
PPD: post partum depression
TNHD: Thai Nguyen provincial health department
VPS: Phan Vietnamese psychiatric scale
